# Evolution of neuroimaging findings in angioinvasive cerebral aspergillosis in a pediatric patient with leukemia during long-term observation

**DOI:** 10.1186/s12879-023-08483-7

**Published:** 2023-11-17

**Authors:** Ali Amanati, Mehrzad Lotfi, Babak Abdolkarimi, Arian Karimi Rouzbahani, Golnaz Mahmoudvand

**Affiliations:** 1 Professor Alborzi Clinical Microbiology Research Center, Shiraz University of Medical Sciences, Shiraz, Iran; 2 Infection Control Unit, Amir Oncology Hospital, Shiraz, Iran; 3 Medical Imaging Research Center, Department of Radiology, Shiraz University of Medical Sciences, Nemazee Hospital, Nemazee Sq., Zand St., Shiraz, 7193613311 Iran; 4 Pediatric Hematology-Oncology, Lorestan University of Medical Sciences, Khorramabad, Iran; 5 Student Research Committee, Lorestan University of Medical Sciences, Anooshirvan Rezaei Sq., Khorramabad, 6814713115 Lorestan Iran; 6 USERN Office, Lorestan University of Medical Sciences, Khorramabad, Iran

**Keywords:** *Aspergillus fumigatus*, Cerebral aspergillosis, Computed tomography (CT), Magnetic resonance imaging, Neuroimaging

## Abstract

The central nervous system is one of the most common sites of aspergillosis involvement in immunocompromised people, just after sinopulmonary infections. Neuroimaging modalities are crucial for the diagnosis of cerebral aspergillosis (CA). Here, we describe a rare case of concurrent mixed aspergillosis infection with *Aspergillus fumigatus* and *Aspergillus niger* in a 2-year-old leukemic boy. The first neuroimaging finding, which was followed by focal seizures, was recognized as extensive cerebral hemorrhage in the absence of thrombocytopenia and coagulopathy. As the patient survived for more than 4 months after diagnosis, we were able to perform a neuroimaging evaluation during long-term observation. In serial neuroimaging studies, a secondary fungal abscess was observed at the site of hemorrhagic infarctions. Finally, the patient died from bacterial sepsis. In this case study, we try to categorize the neuroimaging findings of CA into distinct phases to better understand how CA changes over time.

## Introduction

The central nervous system (CNS) is one of the most important organs affected by *Aspergillus* dissemination [1].

Invasive CNS aspergillosis typically manifests with non-specific clinical signs and symptoms (like seizures or stroke-like symptoms), with or without a fever. CNS aspergillosis may be developed without primary pulmonary infection. Analysis of the cerebrospinal fluid (CSF) is typically only minimally abnormal. It is quite rare to recover the fungal pathogen from the CSF [2].

Cerebral aspergillosis (CA) is mainly caused by the hematogenous dissemination of *Aspergillus* from the lungs; however, it can be a result of invasive sinus involvement [1, 3].

Hematogenous dissemination or direct spread from the paranasal sinuses could be linked to meningeal enhancement, empyema, cerebral abscess, mycotic aneurysms, hemorrhagic lesions, and infrequently stroke [4–6].

The invasive nature of *Aspergillus* within the walls of the larger parent arteries to which it has disseminated hematogenously is most likely what accounts for the apparent predilection of CNS aspergillosis for perforating arterial distributions [5].

CA is usually seen in immunocompromised patients, particularly those with hematologic malignancies. The main risk factors for invasive CA are intense chemotherapy, bone marrow transplantation (BMT), and corticosteroid therapy [1, 6]. Recent advancements in neuroimaging techniques have raised researchers’ attention toward CA afresh [7]. CA commonly usually is fatal even with proper medical antifungal treatment; however, neurosurgical intervention may improve outcomes [8]. An individualized approach should be used when treating patients with refractory or progressive invasive cerebral aspergillosis, considering both the local aspergillus infection epidemiology and the frequency and severity of the infection [9–13].

For example, patients with *A. fumigatus* related aspergillosis may be more likely to experience voriconazole monotherapy failure if the voriconazole MIC is intermediate or resistant (≥ 2 mg/L) or suspected. So, combination therapy with an echinocandin or liposomal amphotericin B should be considered for those with poor clinical response or critical organ involvement [14, 15]. Combination antifungal approaches may be life-saving as a salvage therapy in severely immunosuppressed cancer patients [16].

Depending on the age of the lesion and the patient's immunologic status, cerebral aspergillosis may display different features on neuroimaging. To improve the patient's outcome, it is crucial to diagnose CA as early as possible [2]. We hereby report a rare case of concomitant pulmonary aspergillosis with secondary cerebral involvement in a pediatric leukemic patient with a focus on his neuroimaging findings over long-term observation.

## Case presentation

A 2-year-old boy was referred to the tertiary care pediatric oncology hospital, Shiraz University of Medical Sciences, Iran, due to pallor and fatigue. Laboratory tests revealed anemia and thrombocytopenia. Bone marrow aspiration/biopsy confirmed the diagnosis of acute lymphoblastic leukemia (ALL) and induction chemotherapy, as well as prophylactic trimethoprim/sulfamethoxazole and liposomal amphotericin, were initiated. Galactomannan (GM) tests were run twice a week to look into breakthrough invasive fungal infections. Three weeks after starting intensive chemotherapy, the galactomannan test revealed a positive result about 2 weeks after the start of antifungal prophylaxis (an optical density (OD) index of 0.511 (Platelia™ Aspergillus EIA, sera with an index of ≥ 0.50 considered to be positive for GM antigen). On spiral computed tomography (CT) scan, a pulmonary opacity was observed in the right middle lobe, and intravenous voriconazole was started accordingly (6 mg/kg/dose q/12 h for the first two doses as a loading dose which continued with 4 mg/kg/dose q/12 h as a maintenance dose with regular therapeutic dose monitoring [TDM]). Voriconazole trough levels were maintained within therapeutic levels during treatment. Diagnostic bronchoalveolar Lavage (BAL) was done and *Aspergillosis fumigatus* was recovered from BAL culture on Sabouraud dextrose agar (SDA) (Merck, Germany). The minimum inhibition concentrations (MIC) for caspofungin, voriconazole, and posaconazole were 0.032 mg/l, 0.25 mg/l, 0.032 mg/l, and 0.125 mg/l, respectively.

**Fig. 1:**
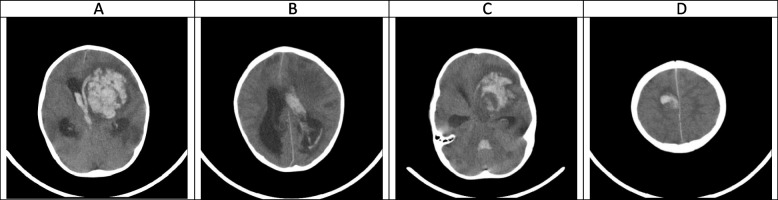
(the brain CT SCAN without contrast, the first imaging study [day 0]). **A** A large heterogeneous hyperdense area seen within the left frontotemporal lobes and associated with marked surrounding edema formation, suggestive of acute hemorrhage and causing significant pressure effect on the left lateral and anterior aspect of third ventricle, resulting marked shift of midline structures to the right side. Also note significant hyperdense material within lateral and third ventricles, suggestive of intraventricular hemorrhage **B** Sign of dilatation of lateral ventricles is seen, suggestive of associated hydrocephalus formation which is secondary to intraventricular hemorrhage and resulting communicating type of hydrocephalus. **C** Signs of two faint rounded hypodense lesions are seen within both cerebellar hemispheres along with other ones at the different parts of both cerebral hemispheres (not shown here) suggestive of early abscess formation. Sign of acute hemorrhage within fourth ventricle is also seen. **D** Sign of another smaller hyperdense lesion at the high convexity of the right parietal lobe also is seen, again suggestive of acute hemorrhage

**Fig. 2:**
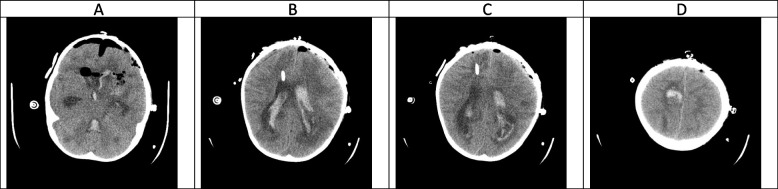
(the brain CT SCAN without contrast, day 1). **A** Significant air within the intracranial cavity, which is due to craniotomy. Faint hypodense lesions which were seen in the previous study are seen again in this study. Multiple air bubbles are seen at the site of the hemorrhagic lesion in the left frontotemporal lobe which is again due to the recent operation and resection of the mentioned lesion. Sign of acute hemorrhage is also seen within the third and fourth ventricles. **B** and **C** shadow of an external ventricular drain (EVD) seen traversing the right frontal lobe and entering the right lateral ventricle. Significant hemorrhage is seen within the lateral ventricles. The midline shift has been improved after the operation. **D** A hemorrhagic lesion is seen at the high convexity of the right frontoparietal junction without significant changes compared with the previous study

**Fig. 3:**
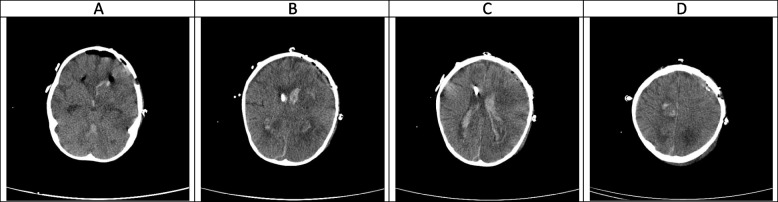
(the brain CT SCAN without contrast, day 3). **A-C** The degree of intracranial and intraparenchymal air,as well as the edema and post-operation changes of the left frontotemporal lobe decreased, however significant intraventricular hemorrhage is again seen, but with no sign of associated hydrocephalus, suggestive of the well-functioning EVD. **D** hemorrhagic lesion of high convexity of the right frontoparietal junction is partially improved

**Fig. 4:**
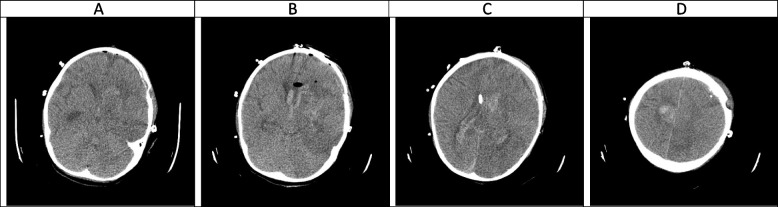
(the brain CT SCAN without contrast, day 7). **A-D** The most of the post-operation changes including intraventricular and intraparenchymal air as well as parenchymal edema mainly disappeared at this time suggestive of improvement of post-operation changes (only minimal intraparenchymal, intraventricular and extra-axial air bubble is seen). A hyperdense rim seen around the left frontal lobe is suggestive of the remnant of post-operative subdural hematoma. Although some degree of intraventricular hemorrhage still could be seen, but no hydrocephalus is found suggestive of the well-functioning EVD. A remnant of hemorrhagic lesion of high convexity of the right frontoparietal junction is seen. Mentioned hypodense lesions within the cerebellar hemispheres and both cerebral hemispheres are faint in this study which can be due to some degree of improvement

**Fig. 5:**
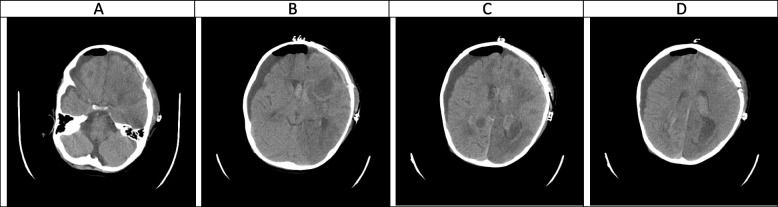
(the brain CT SCAN without contrast, day 9). **A-D** absent right intraventricular EVD is associated with some air within the intracranial cavity and also subdural fluid accumulation around the right cerebral hemisphere, which is due to a recent operation for EVD removal. No hydrocephalus is seen, however, sign of some amount of intraventricular hemorrhage is seen at present. Mild hemorrhage and edema seen at the site of mentioned large left frontotemporal lesion. A hypodense (abscess) lesions which seen within the cerebral hemispheres again seen in this study

**Fig. 6:**
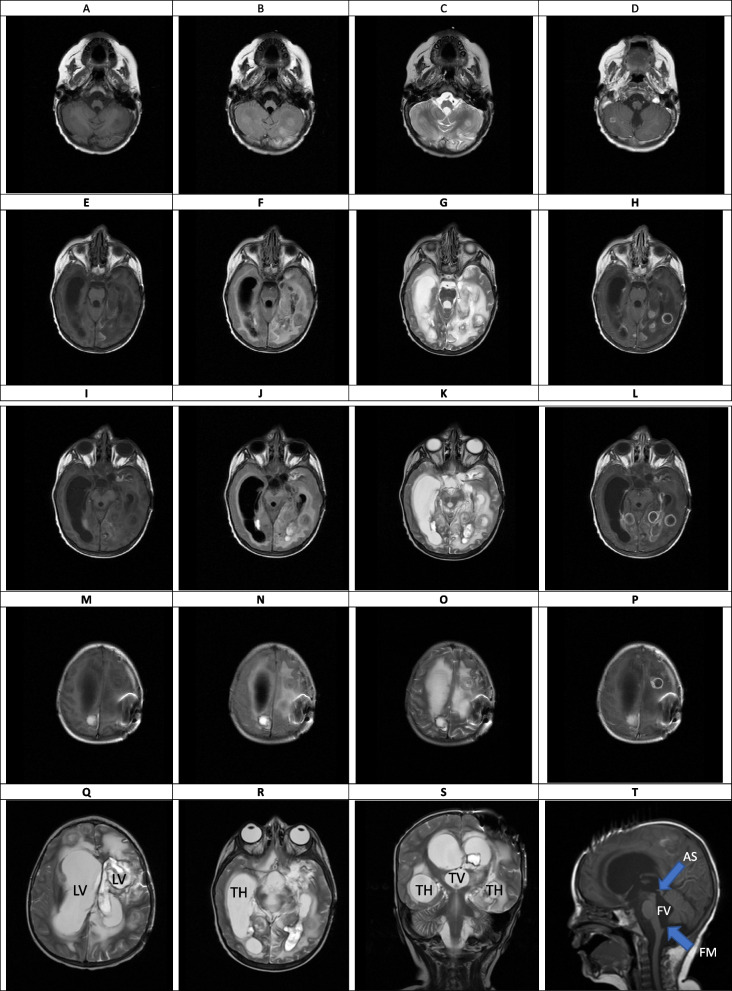
(the Brain MRI Without and with contrast, day 21). **A-T** Multiple varying size ring-like enhancing lesions within the different parts of the white matter of both cerebral and cerebellar hemispheres (some of them representing mentioned hypodense lesion within the previous CT SCAN) suggestive of multiple abscess formation. Sign of significant enhancement of ependymal layer of trigone, occipital and temporal horns of left and lesser degree right lateral ventricles is seen as suggestive of ventriculitis. Sign of marked dilatation of lateral ventricles (including temporal horns) as well as third and fourth ventricles and also aqueduct of *Sylvius* and foramen *Magendie*, all suggestive of communicating type of hydrocephalus (which is most likely secondary to the previous intraventricular hemorrhage and also ventriculitis, obstructing pores of subarachnoid spaces). LV: lateral ventricle, TH: temporal horn of lateral ventricle, TV: third ventricle, FV: fourth ventricle, AS: aqueduct of *Sylvius*, FM: foramen *Magendie*

**Fig. 7:**
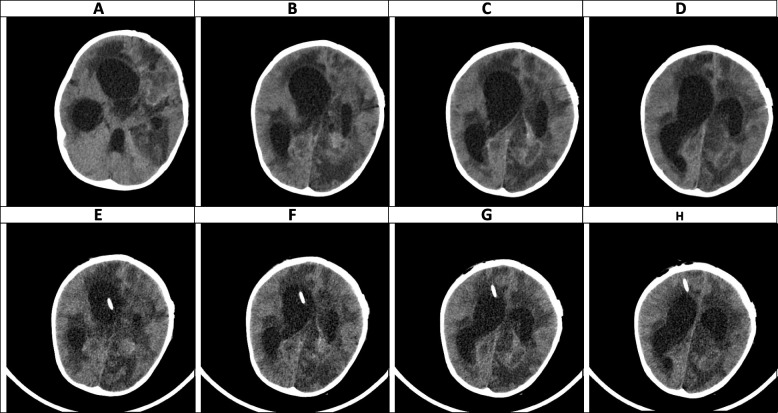
(the Brain CT SCAN without contrast, day 25). **A-D** (before EVD insertion) and **E–H** (after EVD insertion). **A-D** Sign of severe hydrocephalus associated with some shift of midline structures seen to the left side. Signs of multiple hypodense lesions seen at the different parts of the brain parenchyma which is due to mentioned abscess formations. **E–H** Except for mentioned findings in the previous CT SCAN, shadow of a EVD is seen traversing the right frontal lobe and entering the right lateral ventricle, however, the hydrocephalus is persisted which can be due to doing CT SCAN just after EVD insertion rather than EVD malfunction

**Fig. 8:**
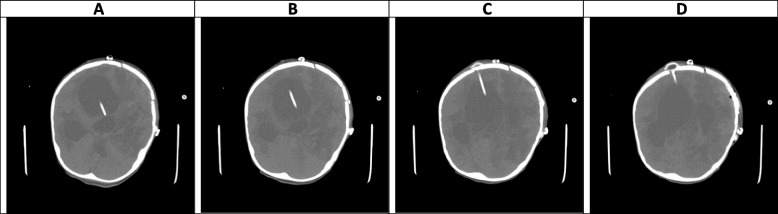
(the Brain CT SCAN without contrast, day 44). **A-D** All of the mentioned findings at the previous CT SCAN are present at this study, including significant hydrocephalus. It means either some degree of EVD malfunction or severity of hydrocephalus

A week after starting antifungal therapy, the patient experienced a focal seizure episode which was controlled by intravenous phenytoin. A brain CT scan was performed which revealed a large heterogeneous hyperdense area within the left frontotemporal lobes in favor of massive intracranial hemorrhage (Fig. 1). Craniotomy was done by a pediatric neurosurgeon with an external ventricular drain (EVD) insertion in the right lateral ventricle and caspofungin was added to his antifungal regimen (70 mg/m^2^ on day 1 and then 70 mg/m^2^ daily). Serial neuroimaging studies were requested by the neurosurgeon and responsible physician according to the patient’s clinical status for a better evaluation of the patient's condition to determine the course of bleeding and prepare the best treatment strategy (Figs. 2, 3, 4 and 5). However, because CT scans and MRIs did not perform in our center, and the neuroimaging studies should be done in other centers (e.g., Nemazee Hospital), we have a limited number of brain magnetic resonance imaging (MRI) and computed tomography (CT) scan was the main diagnostic modality for patient's monitoring. A brain MRI was conducted 18 days following the initial brain CT scan because of uncontrolled seizures which revealed multiple abscess-like lesions (Fig. 6). The images show severe dilatation of lateral ventricles (including temporal horns) as well as third and fourth ventricles and also the aqueduct of *Sylvius* and the foramen of *Magendie*, all suggestive of severe “communicating” or recently named “non-communicating extra-ventricular” type of hydrocephalus [17, 18] (Fig. 6E-H and Q-T, and Fig. 10M-P). Uncontrolled seizures were stopped by the addition of levetiracetam and clonazepam. Corticosteroids and acetazolamide were prescribed to reduce cerebrospinal fluid flow and management of hydrocephalus [19]. Additional EVD and *Ommaya* reservoir was placed by a neurosurgeon (Figs. 7 and 8). *Aspergillus Niger* was identified by SDA on the brain tissue. The MIC for caspofungin, voriconazole, posaconazole, and itraconazole were 0.032 mg/l, 0.5 mg/l, 0.032 mg/l, and 0. 25 mg/l. Also, aspergillosis polymerase chain reaction was reported positive on brain tissue. Nucleic acid was extracted by Invisorb Spin Blood Kit (Stratec, Germany), and Real-time PCR for *Aspergillus* (Primerdesign, United Kingdom) tests were performed according to the manufacturer’s procedure.

**Fig. 9:**
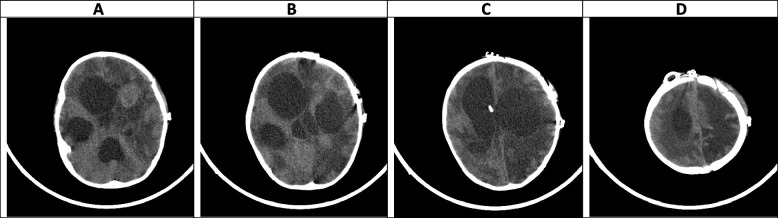
(the Brain CT SCAN without contrast, day 65). **A-D** All of the findings at the previous two CT scans are again present, including significant hydrocephalus, again suggestive of either EVD malfunction or severity of hydrocephalus. Diffuse hypodense areas seen within the brain parenchyma suggestive of brain edema. The hemorrhage of mentioned largest of left frontotemporal lesions is significantly improved at this CT SCAN (the only remnant of hemorrhage is seen)

**Fig. 10:**
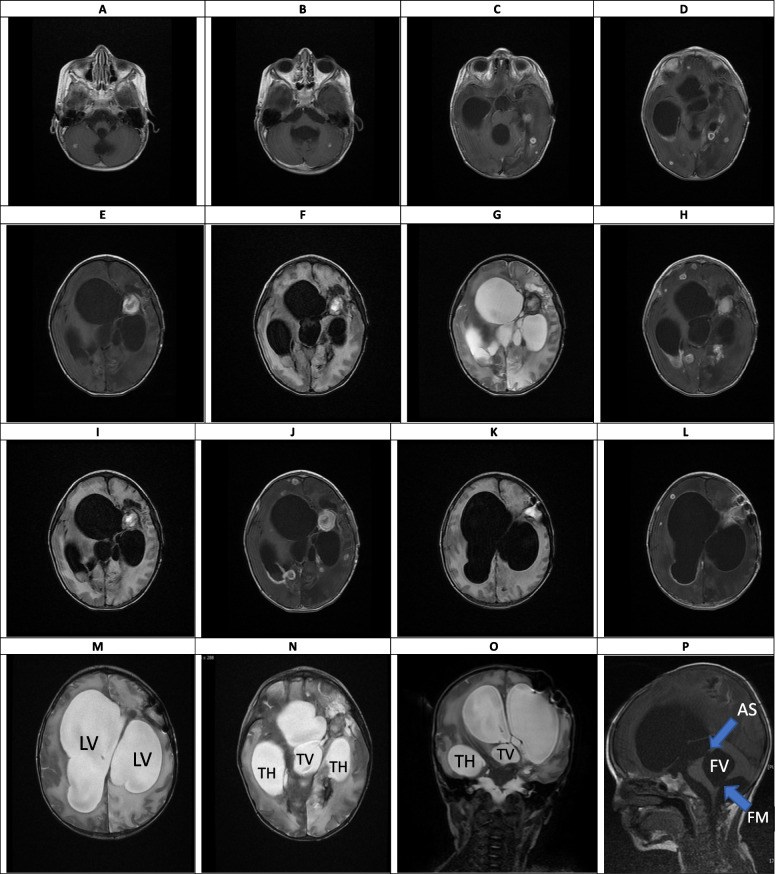
(the Brain MRI Without and with contrast, day 73). **A-P** Sign of multiple ring-like enhancing lesion is seen at the different parts of the brain parenchyma including cerebellar hemispheres associated with surrounding edema formation suggestive of multiple abscess formation (**A-H**). Marked enhancement of ependymal layer of the posterior aspect of the right lateral ventricle (at the level of occipital horn and trigone) and also with a lesser degree at left lateral ventricle is seen which are suggestive of diffuse ventriculitis. Some acute hemorrhage is seen at the site of mentioned largest lesion of the left frontotemporal lobe (**I-L**). Despite of presence of EVD within the right lateral ventricle (not shown here), severe communicating type of hydrocephalus associated with a midline shift to the left side is seen, again suggestive of EVD malfunction or severity of hydrocephalus. Sign of severe dilatation of lateral ventricles (including temporal horns) as well as third and fourth ventricles and also aqueduct of *Sylvius* and foramen *Magendie*, all suggestive of communicating type of hydrocephalus (**M-P**). LV: lateral ventricle, TH: temporal horn of lateral ventricle, TV: third ventricle, FV: fourth ventricle, AS: aqueduct of *Sylvius*, FM: foramen *Magendie*

**Fig. 11:**
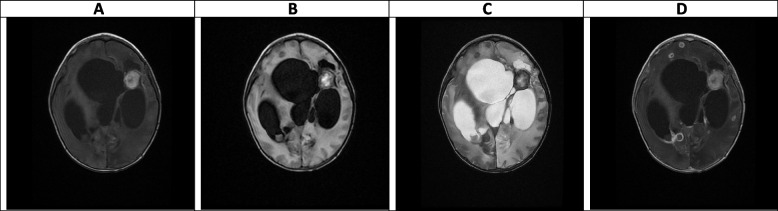
(the Brain MRI Without and with contrast, day 93). **A-D** Most of the mentioned findings in the previous study are seen without significant changes including abscess formation, hemorrhage, sign of ventriculitis, and associated hydrocephalus with midline shift

**Fig. 12:**
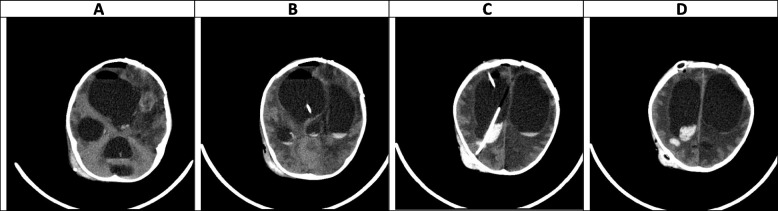
(the Brain CT SCAN without contrast, day 102). **A-D** Some air anterior to the right cerebral hemisphere and within the right lateral ventricle is seen, which is due to a recent operation. shadow of two EVDs is seen traversing the cerebral hemisphere and entering the right lateral ventricle. Some hemorrhage is seen along the path of the posterior EVD which is due to a recent operation. Also, some hemorrhage is seen within the ventricular system which can be due to recent EVD insertion and partly due to the extension of previous hemorrhagic lesions to the ventricular system. Despite the presence of mentioned EVD, sign of the severe communicating type of hydrocephalus is seen, associated with a mild shift of midline structures to the left side. Also sign of previous hypodense lesions and associated diffuse edema formation within the white matter is again seen in this study

Bacterial CSF cultures were negative in repeated samplings. Interventricular amphotericin B deoxycholate (0.5 mg 3 times per week via Ommaya reservoir) and systemic liposomal amphotericin (5 mg/kg/day) were added to his antifungal regimen because of poor clinical response. The patient received triple combination antifungal treatment (voriconazole, caspofungin, and liposomal amphotericin) during his admission course without any side effects. The evolution of the patient’s neuroimaging studies is shown in Figs. 1, 2, 3, 4, 5, 6, 7, 8, 9, 10, 11 and 12. The patient did not experience other invasive organ involvement by aspergillosis. The patient survived for about 4 months without clinical improvement and finally died due to septic shock.

## Discussion

*Aspergillus* is a ubiquitous fungus commonly found in soil and decomposing vegetation. The leading species causing invasive disease are supposed to be *A. fumigatus* complex. Given the increasing incidence of conditions leading to immunosuppression, the burden of invasive aspergillosis is rising [20]. The clinical manifestations of CA are indefinite with the most common presentations being headache, focal neurological deficits, and fever [21]. Despite extensive characterizations of the neuroimaging features of CA and even a few comparative reports versus pyogenic abscesses (including tubercular abscesses) being published, studies on the evolution of CA over time is scarce. The findings of this study provide a better understanding of the neuroimaging aspects of CA over time [22].

In imaging evaluation, CA may be manifested with solitary or multiple ring-enhancing lesions along with striking inflammation and vasogenic edema. Brain abscess is found in 70.2% of the cases but other neuroimaging findings such as cerebral infarction, ventriculitis, and aneurysm are also diagnostic [7, 21, 23]. In rare cases, it may manifest as a space-occupying lesion which can lead to misdiagnosis [24]. CA progress from cerebritis to capsular stages, similar to bacterial abscesses. Classically, brain abscess development can be divided into 4-stages. Early cerebritis (1–4 days), late cerebritis (4–10 days), early capsule formation (11–14 days), and finally late capsule formation (> 14 days) [25].

The basal ganglia and deep white matter are frequently affected by non-enhancing fungal cerebritis. It is usual to find peripheral rim enhancement in mature abscesses [26]. Brain CT scans and MRI are substantial imaging modalities for the diagnosis of CA. MRI is considered the standard diagnostic modality with T1-weighted, T2-weighted, FLAIR, T2^*^, Diffusion-Weighted Imaging (DWI), and T1W after gadolinium injection being the required sequences. On post-contrast imaging, the CA are shown as well-defined rim enhancement lesions that are hypointense on T1-weighted images and hyperintense on T2-weighted images. The apparent diffusion coefficient (ADC) values of the restricted and nonrestricted portions of fungal abscesses are remarkably varied on DW images [22, 27–29].

On T1-weighted images, the intracavitary projections from the wall of fungal abscesses are often isointense to hypointense, and on T2-weighted images, hypointense (both without post-contrast enhancement) [30]. These projections, which were not observed in the pyogenic and tubercular abscesses, are thought to be a differentiating characteristic of fungal abscesses. The wall and the projections both showed restriction of diffusion on DWI (low ADC), while the abscess core itself did not (high ADC) [22, 30]. Compared to pyogenic or tubercular abscesses, fungal abscesses are more likely to have a crenated margin. Satellite lesions are frequently seen in the surroundings of the fungal abscesses [22]. The aspergillus-derived elastase enzyme could cause vasculopathy and accounts for the angio-invasive features of CA. The hyphae invade the vessels and destroy the vessel walls, which causes aneurysm formation that may rupture and lead to serious bleeding. Also, the lumen gets completely blocked by the hyphae leading to ischemic infarction [31]. Initial neurological symptoms in our patient appeared after a massive CNS hemorrhage shortly after pulmonary involvement. In serial neuroimaging studies, a secondary fungal abscess was observed at the site of hemorrhagic infarctions. This event is extremely rare but has been reported previously [32]. The differentiation of the four classic stages of brain abscess could not be identified in our patient because extensive bleeding was the first CA presentation. Instead, we attempted to categorize the neuroimaging findings in our case study into three discrete phases.

### Hyper-acute phase, the first 2 weeks (Figs. 1, 2, 3, 4 and 5)

CA manifests with certain characteristics in different sequences of MRI in the hyper-acute phase. It is commonly associated with a T1W hypo/isointense signal, a T2W hypointense signal, and homogenous enhancement on post-gadolinium T1W [23, 33]. However, Tempkin et al. reported a mas with heterogeneous signal intensity and vasogenic edema on T1W of a CA patient in the hyper-acute phase [34]. Furthermore, Pollack et al. observed an isointense signal along with some regions of hypointensity on T2W [35]. In DWI, heterogeneity of diffusion has been detected in the cerebritis stage (early and late) [28]. Although some case series have revealed uniform DWI restriction at the late capsular stage, other studies have not found evidence of diffusion restriction. This difference might depend on the abscess' cavity viscosity during the late capsular stage (presence or absence of inflammatory cells and fungal hyphae which can induce uniform restriction of diffusion) [22]. In this case, a large hyperdense lesion was discovered in the hyper-acute phase accompanied by marked surrounding edema formation and a shift of midline structures. A sign of dilatation of the lateral and 3rd ventricle resulting in communicating type of hydrocephalus also could be evident. Some faint rounded hypodense lesions were seen within both cerebellar and cerebral hemispheres suggestive of early abscess formation. Signs of intraventricular hemorrhage were found in this phase. By the end of this phase, hydrocephalus had improved following repeated surgical procedures, including an open surgical approach and external ventricular drain (EVD) insertion (Figs. 1, 2, 3, 4 and 5). 

In agreement with our report, CA had manifested with massive cerebral hemorrhage and hemorrhagic infarction in this phase. Also, non-communicating hydrocephalus and ventriculitis could be observed on brain CT scan images in the hyper-acute phase [8]. Rarely, CA might be presented with a homogenously contrast-enhancing lesion on MRI in this phase [31].

### Intermediate phase, week 3- 9 (Figs. 6, 7, 8 and 9)

The main findings in the intermediate phase in this case were a massive hemorrhagic lesion with some ring-like enhancement lesions suggestive of early reactivation, a sign of significant enhancement of the ependymal layer of the trigone, in addition to enhancement of the occipital, temporal horns, and lateral ventricles that suggestive of early ventriculitis. Also, a severe communicating type of hydrocephalus which is most likely secondary to the previous intraventricular hemorrhage, ventriculitis, obstructing pores of subarachnoid spaces, and foramen *Monroe* were detected in this phase. Decreased brain edema was found at the end of this phase (Figs. 6, 7, 8 and 9). The brain CT scan in the intermediate phase may reveal edema and midline shift while gadolinium-enhanced MRI may detect multiple abscesses formation [8]. Lee et al. found a low-signal intensity on T1W and a high-signal intensity on T2W in a CA patient in the intermediate phase. In the gadolinium-enhanced study, several nodular-enhancing lesions with or without focal necrosis could be observed [36].

### Late phase, after week 10 (Figs. 10, 11 and 12)

The data on neuroimaging findings in the late phase is limited. In our case, resolving hemorrhagic lesions, multiple ring-like enhancing lesions, ventriculitis, and communicating type of hydrocephalus were the main findings in this phase (Figs. 10, 11 and 12). Negoro et al. observed mycotic aneurysm as the neurological manifestation of CA in this phase [37]. In the case reported by Gayol et al., the lesion developed a necrotic center in the late phase. Following a surgical procedure, the patient developed an infarct and subsequently communicated hydrocephalus [31].

## Conclusion

Since CA is a potentially deadly condition, there is a scarcity of data on serial neuroimaging findings of patients during long-term assessment. We observed intraventricular hemorrhage with communicating type of hydrocephalus as neurological findings in the hyper-acute phase, and multiple abscess formation and ventriculitis in intermediate and late phases. With new therapeutic approaches, the survival rate of CA patients may improve, adding more knowledge to late-phase imaging findings.

## Data Availability

The datasets used during the current study are available from the corresponding author upon reasonable request.

## Abbreviations

CA: Cerebral aspergillosis
CT scan: Computed tomography scan
CNS: Central nervous system
ALL: Acute lymphoblastic leukemia
MRI: Magnetic resonance imaging
DWI: Diffusion-Weighted Imaging
ADC: Apparent diffusion coefficient
